# Hospital presentations for self-poisoning during COVID-19 in Sri Lanka: an interrupted time-series analysis

**DOI:** 10.1016/S2215-0366(21)00242-X

**Published:** 2021-10

**Authors:** Duleeka Knipe, Tharuka Silva, Azra Aroos, Lalith Senarathna, Nirosha Madhuwanthi Hettiarachchi, Sampath R Galappaththi, Matthew J Spittal, David Gunnell, Chris Metcalfe, Thilini Rajapakse

**Affiliations:** a Population Health Sciences, Bristol Medical School, University of Bristol, Bristol, UK; b South Asian Clinical Toxicology Research Collaboration, Faculty of Medicine, University of Peradeniya, Peradeniya, Sri Lanka; c Department of Psychiatry, Faculty of Medicine, University of Peradeniya, Peradeniya, Sri Lanka; d Department of Health Promotion, Rajarata University of Sri Lanka, Mihintale, Sri Lanka; e Toxicology Unit, Teaching Hospital, Peradeniya, Sri Lanka; f Centre for Mental Health, Melbourne School of Population and Global Health, University of Melbourne, Melbourne, VIC, Australia; g National Institute of Health Research Biomedical Research Centre, University Hospitals Bristol NHS Foundation Trust, Bristol, UK

## Abstract

**Background:**

There is widespread concern over the impact of public health measures, such as lockdowns, associated with COVID-19 on mental health, including suicide. High-quality evidence from low-income and middle-income countries, where the burden of suicide and self-harm is greatest, is scarce. We aimed to determine the effect of the pandemic on hospital presentations for self-poisoning.

**Methods:**

In this interrupted time-series analysis, we established a new self-poisoning register at the tertiary care Teaching Hospital Peradeniya in Sri Lanka, a lower-middle-income country. Using a standard extraction sheet, data were gathered for all patients admitted to the Toxicology Unit with self-poisoning between Jan 1, 2019, and Aug 31, 2020. Only patients classified by the treating clinician as having intentionally self-poisoned were included. Data on date of admission, age or date of birth, sex, and poisoning method were collected. No data on ethnicity were available. We used interrupted time-series analysis to calculate weekly hospital admissions for self-poisoning before (Jan 1, 2019–March 19, 2020) and during (March 20–Aug 31, 2020) the pandemic, overall and by age (age <25 years *vs* ≥25 years) and sex. Individuals with missing date of admission were excluded from the main analysis.

**Findings:**

Between Jan 1, 2019, and Aug 31, 2020, 1401 individuals (584 [41·7%] males, 761 [54·3%] females, and 56 [4·0%] of unknown sex) presented to the hospital with self-poisoning and had date of admission data. A 32% (95% CI 12–48) reduction in hospital presentations for self-poisoning in the pandemic period compared with pre-pandemic trends was observed (rate ratio 0·68, 95% CI 0·52–0·88; p=0·0032). We found no evidence that the impact of the pandemic differed by sex (rate ratio 0·64, 95% CI 0·44–0·94, for females *vs* 0·85, 0·57–1·26, for males; p_interaction_=0·43) or age (0·64, 0·44–0·93, for patients aged <25 years *vs* 0·81, 0·57–1·16, for patients aged ≥25 years; p_interaction_=0·077).

**Interpretation:**

This is the first study from a lower-middle-income country to estimate the impact of the pandemic on self-harm (non-fatal) accounting for underlying trends. If the fall in hospital presentations during the pandemic reflects a reduction in the medical treatment of people who have self-poisoned, rather than a true fall in incidence, then public health messages should emphasise the importance of seeking help early.

**Funding:**

Elizabeth Blackwell Institute University of Bristol, Wellcome Trust, and Centre for Pesticide Suicide Prevention.

**Translations:**

For the Sinhalese and Tamil translations of the abstract see Supplementary Materials section.

## Introduction

The COVID-19 pandemic and the associated public health measures, including lockdowns, intended to limit exposure to the virus have had a huge effect on populations worldwide. Being in lockdown appears to have contributed to feelings of isolation, experiences of domestic violence, financial and job insecurity, and social stigma associated with COVID-19, all of which can contribute to an increased risk of suicidal behaviour.^1^

Despite potential concerns over the negative impact of the pandemic on suicide and self-harm, emerging evidence from many countries suggests that during the early months after the COVID-19 outbreak, there was either no change or a reduction in suicide deaths.^2^ This evidence, however, needs to be interpreted with caution given that almost no data from low-income and middle-income countries (LMICs) were included in this study. The impact of the pandemic on suicide and self-harm in LMICs is unclear.^3^ Many LMICs have enforced similar lockdown restrictions to those put in place in high-income countries. Most LMICs, however, do not have the resources to provide adequate mitigating measures (eg, financial support), and many people living in these countries are part of the informal economy; thus, their income is less stable. In addition, the restrictions imposed are likely to have indirect effects that could detrimentally affect progress made in the past with other causes of death (eg, infant mortality^4^, ^5^).

Current evidence for the effects of the pandemic on suicide and self-harm in LMICs has largely been restricted to media reports or case series,^3^ which have reported mixed findings. Studies from India and Nepal have suggested an increase in deaths by suicide and in the number of individuals presenting to hospitals with self-harm in the pandemic period.^6^, ^7^ However, these studies compare a period before the pandemic to a period during the pandemic and do not account for any underlying trends. Evidence from Peru, Serbia, and Turkey suggests a decline in the number of individuals presenting with self-harm to hospitals and suicide deaths.^8^, ^9^ The study from Peru did account for underlying trends but was restricted to suicide as the outcome.

**Research in context**

**Evidence before this study**

Evidence on the impact of the pandemic on suicide and self-harm largely originates from high-income countries, with limited evidence from low-income and middle-income countries (LMICs), where 80% of suicide deaths occur. We developed a living systematic review that searches the literature daily via PubMed, Scopus, *medRxiv, bioRxiv*, the COVID-19 Open Research Dataset by Semantic Scholar, the Allen Institute for AI, and the WHO COVID-19 database. We used an extensive list of search terms for suicide (eg, “suicid*”), suicidal behaviour (eg, “attempted suicide”), and self-harm (eg, “self-harm*”), in combination with terms for COVID-19 (eg, “coronavirus” OR “COVID*” or “SARS-CoV-2”; appendix 3 p 1). Databases were searched between Jan 1, 2020, and March 23, 2020, with no language restrictions (appendix 3 p 1). As of March 23, 2020, there were 47 articles on LMICs (24 [51%] were based on media reports), with no studies accounting for temporal trends in self-harm, and only one time-series study reporting on the impact of the pandemic on suicide rates in Peru (a middle-income country); this study found a decrease in suicide deaths.

**Added value of this study**

To our knowledge, this study is the first to estimate the impact of the COVID-19 pandemic on individuals presenting to a hospital with self-harm in a LMIC, accounting for underlying trends. During the first 5 months of the pandemic, the number of people presenting to the hospital with self-poisoning reduced by nearly a third in Sri Lanka compared with pre-pandemic levels. This finding appears to be due to a reduction in people presenting with self-poisoning through medicinal overdoses.

**Implications of all the available evidence**

Evidence from high-income countries indicates a reduction in suicide and self-harm during the early months of the pandemic and the findings of this study suggest the same is true in at least one lower-middle income country. Some caution is required in case individuals who had self-harmed were less likely to present to hospital after self-poisoning. Failure to seek treatment might result in adverse consequences of untreated poisoning, including death, and in the lack of appropriate psychiatric assessment and aftercare in this high-risk population. If the fall in hospital presentations during the pandemic reflects a reduction in the medical treatment of people who have self-poisoned—eg, because of their concerns about the risk of contracting COVID-19 while in hospital—rather than a true decline in incidence, then public health messages should emphasise the importance of seeking help early.

In Sri Lanka, self-poisoning is the most common method of non-fatal self-harm in people presenting to hospital,^10^ and accounted for 822 (26%) of 3135 suicide deaths in 2019.^11^ Using hospital record data from a Toxicology Unit in a large tertiary hospital in Sri Lanka, we aimed to determine whether the COVID-19 pandemic resulted in a change in the numbers of individuals presenting with self-harm (by self-poisoning) compared with expected trends and whether there is evidence that the impact of the pandemic differed by sex, age, and type of poison ingested.

## Methods

### Study design

This interrupted time-series analysis was based in Sri Lanka, a lower-middle-income country with a population of 21 million (census 2011), a high rate of suicide, and a rising rate of self-harm.^12^, ^13^ In response to the COVID-19 pandemic, on March 20, 2020, a nationwide continuous curfew was enacted (ie, all individuals had to remain indoors starting from 1800 h on March 20, 2020, until 0600 h on March 23, 2020), which was then extended until April 27, 2020, with brief periods when the curfew was lifted for a few hours on a selected number of days. From April 27, 2020, the rules were changed to allow individuals time outside their homes to allow access to essential services. The initial lockdown periods were enforced by the army and police, and movement was continuously and strictly restricted, except for emergencies and for people working in essential services. By June 28, 2020, all lockdown measures were lifted. A series of additional regional (and some national) measures of varying intensity were then introduced to control the spread of the virus.

Data were collected from the Teaching Hospital Peradeniya in the Kandy district in the Central Province of Sri Lanka. The Kandy district mirrors the demographic profile of the country in terms of the age and sex distribution, with a Sinhalese and Buddhist majority. Teaching Hospital Peradeniya is a tertiary care hospital, with about 900 beds, and provides medical care for about 70–80 000 inpatients and 350 000 outpatients per annum. The Toxicology Unit of the hospital provides treatment for individuals presenting with self-poisoning (both intentional and accidental) from the Central Province and surrounding regions.

### Data collection

A new research register was established in June, 2020, to track the impact of COVID-19 on hospital admissions for self-poisoning in Sri Lanka through routinely collected hospital data. This register included both retrospective (Jan 1, 2019–June 15, 2020) and ongoing collection of hospital admissions for self-poisoning. The start date was chosen to include sufficient time before the pandemic to allow for the modelling of pre-pandemic trends. All individuals who present with poisoning at Teaching Hospital Peradeniya are admitted to the Toxicology Unit for treatment, and there were no changes to poisoning admission procedures because of the pandemic. The physical location of the Toxicology Unit changed within Teaching Hospital Peradeniya, with the previous location being designated a COVID-19 isolation ward on March 26, 2020. This change resulted in the capacity of the ward being reduced to 18 beds from 36. However, the admission policy remained unchanged, such that patients requiring hospital treatment were admitted irrespective of bed availability. Both accidental and intentional poisoning admission numbers were collected from the Toxicology Unit admission books, and the classification of accidental or intentional was decided by the treating clinician. Admission book data and information from bed head tickets were used to identify cases of self-poisoning (ie, intentional self-harm by ingesting poison). A bed head ticket is the medical record of the inpatient and contains all relevant information regarding the patient's stay, including any related history. The overall number of admissions per month were collected from the admission books and used to trace bed head tickets for detailed information regarding admissions (eg, type of poison ingested). Where bed head tickets were unable to be traced, admission books were revisited to collect admission dates. Admission due to accidental poisoning was excluded from the analysis. Using a standard extraction sheet, data were gathered for all self-poisoning cases admitted to the ward between Jan 1, 2019, and Aug 31, 2020. Data were collected by trained clinical research assistants under the supervision of the study authors, who regularly checked the admission books against the data collected from bed head tickets to ensure cases were not missed. The data were extracted by a single research assistant. Data on date of admission, age or date of birth, sex, and poisoning method were collected. Ethnicity data were not available from routine records. Ethical approval for the register was obtained from the Ethics Review Committee at the Faculty of Medicine, University of Peradeniya.

### Data analysis

All data were cleaned and analysed according to a prespecified and registered analysis plan.^14^ Data on the dates of admission were converted to weekly data, with weeks beginning on Sunday and ending on a Saturday. Patient age was calculated using their date of birth (where available) or reported age. On the basis of the recommended cutoff values for defining adolescence and youth, and knowledge of the patient profile of this ward before the pandemic,^15^, ^16^ we categorised patients into young (<25 years) and older (≥25 years) individuals. We categorised poisoning into medicinal, agrochemical, and plant or other on the basis of the toxicological agent. If a combination of poisoning methods were used, these admissions were categorised as plant or other. We further categorised medicinal poisoning cases into analgesic, metabolic, psychiatric, or other drugs.

In the main analysis, we examined changes in numbers of weekly admissions for self-poisoning in the pre-pandemic period (Jan 1, 2019–March 19, 2020) and pandemic period (March 20–Aug 31, 2020) using interrupted time-series analysis. We fitted Poisson regression models with a scale parameter to account for overdispersion. We fitted models for the overall number of presentations by sex and age groups. We used the *fp* function in Stata statistical software (version 16.1) to incorporate longer-term time trends as fractional polynomials in each of these models. We hypothesised that there would be a step change in weekly hospital presentations, and therefore all of our models included a binary coded predictor in the model that represented the pandemic period and included the period when lockdown restrictions were in place. We report the effect of this predictor as a rate ratio with 95% CI. For age-stratified and sex-stratified models, we used the Kandy district population data as an offset term to allow us to make a direct comparison between the strata. To test whether the impact of the lockdown measures differed by age or sex, we fitted interrupted time-series regression models including age or sex (binary variable) as a covariate and formally tested for an interaction. We graphically present weekly changes in self-poisoning admissions overall, and by age group, sex, and poison type.

Our main analysis was restricted to 20 months of data, and therefore we were unable to account for any seasonal trends. As a post-hoc sensitivity analysis, we included an additional binary variable in the main model (ie, changes to overall trends) to test whether the effect observed in 2020 (ie, the pandemic period) was observed in an equivalent period in 2019 (ie, the pre-pandemic period). We assessed whether the inclusion of this binary variable improved the fit of the model by comparing the Akaike information criterion (AIC) for each model.

The main analysis excluded individuals with missing date of admission. To explore whether the exclusion of these patients affected the overall conclusions of the study, in our second sensitivity analysis, we refitted the main model using self-poisoning presentations by month, and specified the pandemic period as starting from April 1, 2020.

As a final post-hoc sensitivity analysis, we fitted two additional models that tested whether changes in data collection or reporting altered the overall conclusions of our results (appendix 3 p 1). As the number of ward beds decreased in the pandemic period, we also collected information on the number of occupied beds in the first 10 weeks after the start of the pandemic (ie, March 20, 2020). We randomly selected one day of the week from each of the first 10 weeks of the pandemic and compared this percentage of occupied beds to the same 10 days in 2019.

### Role of the funding source

The funders of the study had no role in study design, data collection, data analysis, data interpretation, or writing of the report.

## Results

Between Jan 1, 2019, and Aug 31, 2020, 1416 individuals were admitted to hospital for self-poisoning. During data analysis, we noted that for 68 hospital presentations, the date of admission was missing. The proportion of missing bed head tickets was similar in the pre-pandemic and pandemic periods (56 [4·8%] of 1161 *vs* 12 [5·0%] of 240). We were able to obtain date of admission for 53 of these patients (42 [75·0%] of 56 of pre-pandemic and 11 [91·7%] of 12 of pandemic cases) and month of presentation for the remaining 15 patients. However, all other data were missing for these 15 patients, and they were therefore excluded from the main analysis. Of the 1401 patients included, 584 (41·7%) were males and 761 (54·3%) were females, and these data were missing for 56 (4·0%; table) patients.

**Table:** Baseline description of patients admitted to Teaching Hospital Peradeniya (Sri Lanka) with self-poisoning, by pandemic period

				**Pre-pandemic period (n=1161)**	**Pandemic period (n=240)**
Number of people presenting with self-poisoning per week				18 (7)	10 (4)
Patients with bed head ticket data					
	Age, years			23 (18–34)	25 (18–38)
		<25		603 (51·9%)	107 (44·6%)
		≥25		546 (47·0%)	117 (48·8%)
		Missing		12 (1·0%)	16 (6·8%)
	Sex				
		Male		480 (41·3%)	104 (43·3%)
		Female		638 (55·0%)	123 (51·3%)
		Missing		43 (3·7%)	13 (5·4%)
	Time of attendance				
		0000 h to 0759 h		132 (11·4%)	20 (8·3%)
		0800 h to 1559 h		354 (30·5%)	99 (41·3%)
		1600 h 2359 h		629 (54·2%)	109 (45·4%)
		Missing		46 (4·0%)	12 (5·0%)
	Psychiatric assessment				
		Yes		1039 (89·5%)	215 (89·6%)
			Consultant	42 (3·6%)	24 (10·0%)
			Registrar	403 (34·7%)	86 (35·8%)
			Senior registrar	61 (5·3%)	15 (6·3%)
			Medical officer	504 (43·4%)	89 (37·1%)
			Unknown	95 (8·2%)	14 (5·8%)
		No		56 (4·8%)	12 (5·0%)
		Unknown		66 (5·7%)	13 (5·4%)
	Current psychiatric diagnosis				
		Yes		535 (46·1%)	110 (45·8%)
		No		482 (41·5%)	97 (40·4%)
		Unknown		144 (12·4%)	33 (13·8%)
	Case fatality rate* (95% CI)			0·9 (0·3–1·4)	2·5 (0·5–4·5)
	Method of self-poisoning				
		Medicinal		590 (50·8%)	117 (48·8%)
		Agrochemical (pesticide or insecticide)		205 (17·7%)	58 (24·2%)
		Plant or other		324 (27·9%)	54 (22·5%)
		Missing		42 (3·6%)	11 (4·6%)

Data are mean (SD), median (IQR) or n (%) unless otherwise stated. Percentages might not sum to 100% owing to rounding. The pre-pandemic period was defined as Jan 1, 2019, to March 19, 2020, and the pandemic period was defined as March 20 to Aug 31, 2020. 15 patients with missing date of admission were excluded.

* 127 patients had missing outcome data (92 from the pre-pandemic period, and 35 from the pandemic period), and these patients were assumed to be alive.

**Figure 1:**
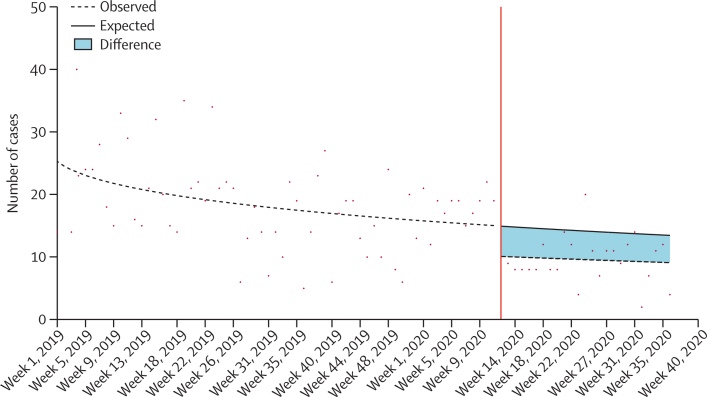
Changes in admissions for self-poisoning before and during the COVID-19 pandemic in Teaching Hospital Peradeniya, Sri Lanka The vertical line indicates the beginning of the national lockdown on March 20, 2020.

**Figure 2:**
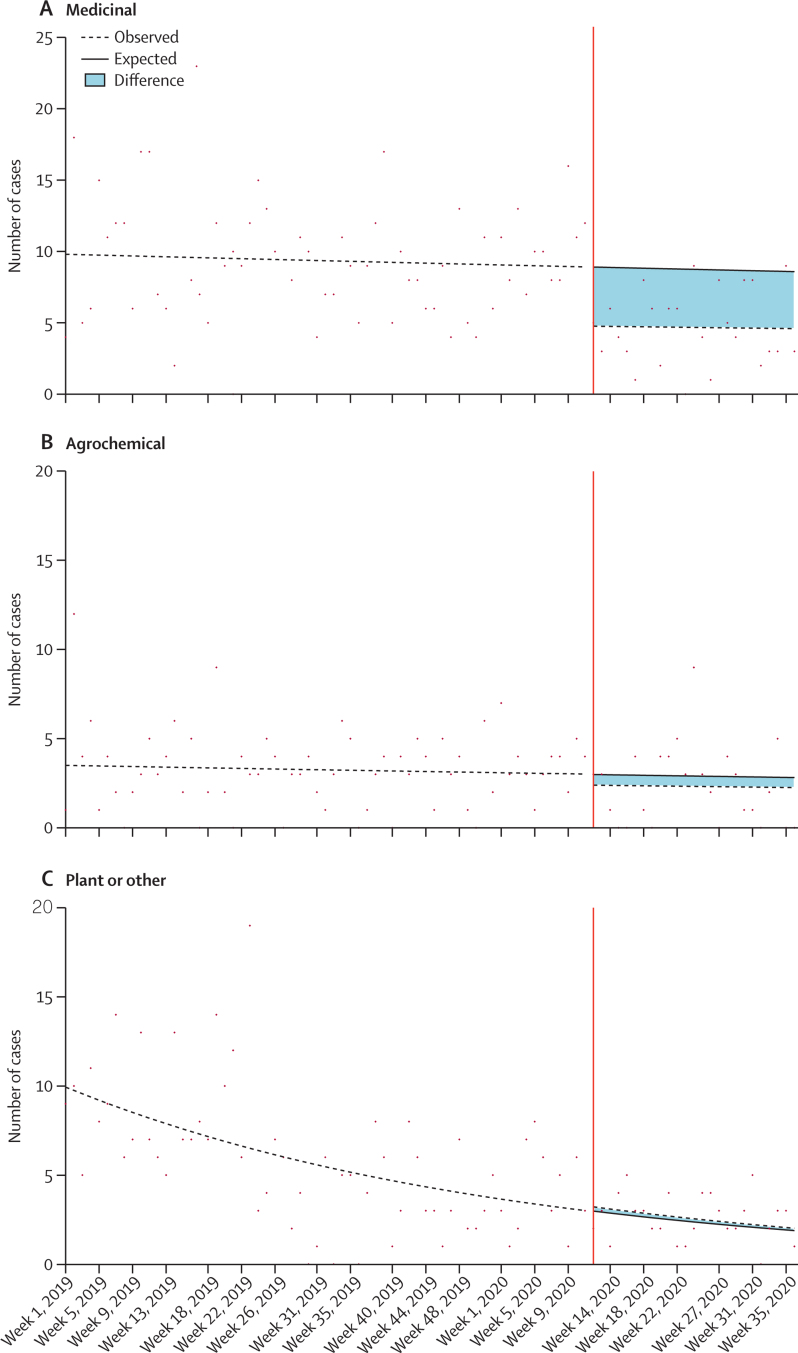
Changes in admissions for self-poisoning by poison type before and during the COVID-19 pandemic in Teaching Hospital Peradeniya, Sri Lanka The vertical line indicates the beginning of the national lockdown on March 20, 2020.

A sudden drop in presentations for self-poisoning occurred at the start of the lockdown period (figure 1); on average, there were more presentations in the pre-pandemic period than in the pandemic period (table). The time-series analysis indicated that there was a 32% (95% CI 12–48) reduction in hospital presentations for self-poisoning during the pandemic period compared with the pre-pandemic period (rate ratio 0·68, 95% CI 0·52–0·88; p=0·0032), and this reduction was independent of a steady long-term reduction in presentations following self-poisoning. 409 (57·9%) of 707 medicinal overdoses were by analgesics; this proportion was largely unchanged in the two periods under study (342 [58·0%] of 590 in the pre-pandemic period *vs* 67 [57·3%] of 117 in the pandemic period). It appears that the drop in overall presentations was driven by a decrease in hospital presentations for medicinal overdoses (figure 2), and the fall in medicinal overdoses appeared to be mostly due to a drop in analgesic poisoning (appendix 3 p 2).

**Figure 3:**
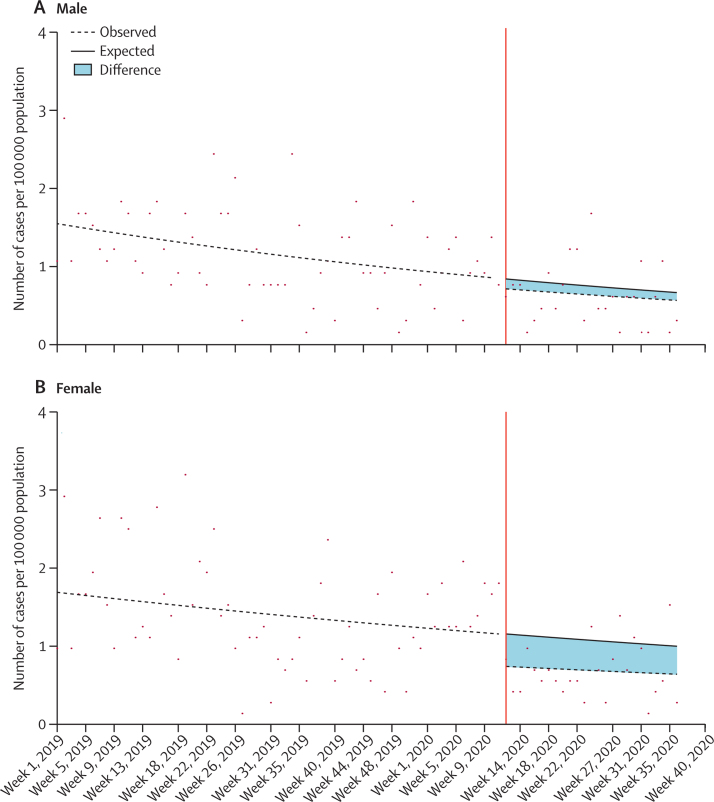
Changes in admissions for self-poisoning by sex before and during the COVID-19 pandemic in Teaching Hospital Peradeniya, Sri Lanka The vertical line indicates the beginning of the national lockdown on March 20, 2020.

There was a reduction in presentations during the pandemic compared with the pre-pandemic period for females (rate ratio 0·64, 95% CI 0·44–0·94; p=0·021) and males (0·85, 0·57–1·26; 0·42; figure 3). The difference in the impact of the pandemic by sex is consistent with chance (p_interaction_=0·43).

**Figure 4:**
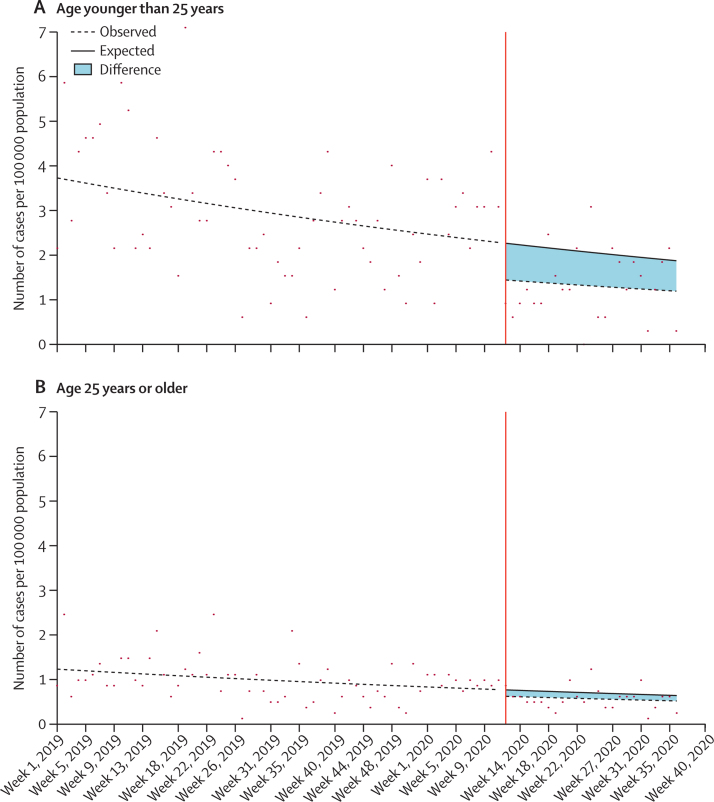
Changes in admission for self-poisoning by age group before and during the COVID-19 pandemic in Teaching Hospital Peradeniya, Sri Lanka The vertical line indicates the beginning of the national lockdown on March 20, 2020.

The median age of patients in the pre-pandemic and pandemic periods was similar, with a slightly higher proportion of patients in the younger age group (<25 years) in the period before the pandemic than during the pandemic (table). Although the reduction was more marked in the younger age group (rate ratio 0·64, 95% CI 0·44–0·93; p=0·18) than in the older age group (0·81, 0·57–1·16; p=0·26; figure 4), there was no statistical evidence that the impact of the pandemic differed by age (p_interaction_=0·077).

We tested whether the inclusion of a binary variable for the same period during 2019 as the pandemic period in 2020 (weeks 12–36) improved the fit of our main model (overall presentations). We found no evidence that the inclusion of this binary variable improved model fit (with variable AIC 6·77; without variable AIC 6·76).

The week of presentation was missing for 15 hospital presentations, with 14 occurring in 52 weeks in 2019, and one in 36 weeks in 2020. The majority of missing data (for 11 [79%] of 14 cases) in 2019 were in the first 6 months. Therefore, we refitted the main model comparing number of overall presentations by month before (Jan 1, 2019–March 31, 2020) and during the pandemic (April 1–Aug 31, 2020). Consistent with our main model, the number of presentations declined by 19% in the pandemic period, although the strength of the association was reduced (rate ratio 0·81, 95% CI 0·60–1·10; p=0·18). Changes in the method of data collection or reporting did not alter the overall conclusions of our results (appendix 3 p 2).

## Discussion

To the best of our knowledge, this study is the first to estimate the impact of the COVID-19 pandemic on people presenting to hospital with self-harm in a low-income or middle-income country, taking into account underlying trends. We found that during the first 5 months of the pandemic, the number of hospital admissions for self-poisoning reduced by nearly a third, mainly due to a reduction in presentations due to overdoses of analgesics.

The findings of this study differ from similar previous investigations in neighbouring countries (India and Nepal), where there are reports of an increase in hospital presentations for self-harm^7^ and suicide.^6^ However, these previous investigations did not take into account pre-pandemic trends.^6^, ^7^ Given that pre-pandemic reports have indicated that the number of people presenting to hospital with self-harm and suicide in India and Nepal have increased over time,^17^, ^18^, ^19^ the reported increases during the pandemic might reflect increases due to other underlying factors, as opposed to an increase related to the pandemic. A previous investigation in another middle-income country (Peru) modelled underlying trends of suicide and indicated a step reduction in suicide during the first few months of the pandemic. This reduction is similar to the findings of suicides in high-income countries.^20^, ^21^, ^22^ Tracking the effects of the pandemic will be key, as emerging evidence from Japan, Vienna, and Puerto Rico suggests that following an initial decline in suicidal behaviour, rates have started to increase.^2^, ^23^

The reduction in hospital presentations for self-poisoning in Sri Lanka during the first few months of the pandemic period could be due to a reduced incidence of self-poisoning as a consequence of increased social integration during times of community-wide disasters.^24^ The absence of data to assess social integration, or community-level mental distress (including community self-harm), means the hypothesis is difficult to test. Evidence in high-income countries suggests that levels of suicidal ideation and self-harming behaviour in the community have not changed during the pandemic period.^25^, ^26^ Reassuringly, the age-adjusted rate of suicide in Sri Lanka in 2020 was slightly lower than the rate in 2019 (13·9 per 100 000 *vs* 14·2 per 100 000 population), with suicide deaths due to poisoning also showing a decline (data available on request from the Department of Police, Sri Lanka).

Previous evidence shows that presenting to hospital with self-poisoning is related to interpersonal conflict,^27^ and that about 40% (50% female; 36% male) of individuals who present to hospital with self-poisoning are current victims of domestic violence.^28^ Given reports of increases in domestic violence and conflicts during the COVID-19 pandemic,^29^, ^30^ it might therefore be reasonable to expect a concurrent rise in self-harm. The absence of a rise, as indicated in our data, might reflect increased proximity of household members during the pandemic period; thus, the opportunity to self-poison might have decreased or access to services after a self-harm incident might have been controlled by perpetrators of domestic violence. In addition, domestic violence and conflicts are often associated with alcohol misuse by male household members in Sri Lanka.^31^ During the early pandemic restrictions, the Sri Lankan Government banned the sale of alcohol. Based on a cross-sectional survey of 2019, the alcohol sale ban appears to have resulted in a reduction of self-reported family problems as a consequence of “husband[s] reducing alcohol use”.^32^ The survey also indicated that 80% of respondents who reported consuming alcohol had reduced their consumption during the pandemic (both legal and illegal).

The lockdown might also have limited access to over-the-counter medicines that could be used to overdose because most pharmacies were closed during the initial nationwide lockdown and travel was severely restricted. Affected individuals might have substituted poisoning with other methods of self-harm.

During the early stages of the pandemic when movement in Sri Lanka was severely restricted and the fear of contracting COVID-19 was high, there might have been an overall reduction in help seeking from hospital services, and an increase in home remedies such as those described by Sirisena for analgesic self-poisoning.^33^ The reduction in hospital presentations for self-poisoning might reflect an overall decline in service use during the pandemic. If self-poisoning in the community has not declined, a reduced willingness to present to hospital might have serious implications for the long-term health of individuals who have ingested poison. If so, increasing awareness about the importance of seeking treatment immediately after self-poisoning should be part of public health messages during the pandemic, while taking care to avoid any inadvertent glamourisation of self-harm behaviours. Further work assessing and tracking the level of community level mental distress is urgently needed, with specific investigations into whether treatment seeking for self-poisoning has changed.

Pre-pandemic research indicates that older individuals who have self-harmed and presented to hospital tend to have more associated psychiatric morbidity, and use more lethal methods, such as agrochemical ingestion.^34^ Our findings suggest that the number of people self-poisoning with agrochemicals did not drop during the pandemic, in contrast to the decline of medicinal overdoses during the same period. The pandemic restrictions did not affect farming activities, and agricultural supplies, including agrochemicals, were still available.^35^ The movement restrictions and closures of centres that collect and distribute produce have disrupted the market for perishable fruit and vegetables, affecting farming livelihoods.^35^ The negative economic impact coupled with access to pesticides might result in increased self-harm and suicide in farming households as the impact of the lockdown measures is realised. Nearly two-thirds of the Sri Lankan work force are part of the informal economy: these individuals are likely to be hardest hit by the negative impact of the pandemic on the national economy as it is a group that is already at highest risk of suicide and self-harm.^36^ Support for these individuals must continue and be increased as needed to prevent suicide and self-harm.

This study has methodological limitations that should be considered when interpreting the findings. First, we tracked the impact of the pandemic only on people presenting to hospital with self-poisoning and it is possible that during the pandemic people substituted self-poisoning with other methods. Individuals who self-harm by other methods might be less likely to present to hospital because the method is more amenable to self-care or associated with a higher case fatality.^37^ Second, the data used in this analysis for presentations before June 1, 2020, were collected retrospectively through admission book and bed head ticket notes review. Whilst every attempt was made to retrieve at least the basic characteristics for each admission, we were unable to do this for a small proportion of cases. The inclusion of these cases might have altered our results, although our sensitivity analysis did not indicate that this would have been the case. Third, given that the physical location of the ward changed within Teaching Hospital Peradeniya after 6 days of the announcement of lockdown in Sri Lanka, we are unable to formally evaluate whether the changes we observed were driven by a reduction in self-poisoning presentations to hospital, or due to a knock-on effect on referrals within the hospital because of the change in location and reduction in ward capacity. It is possible that staff in the outpatient department (synonymous with an emergency department) were less likely to refer patients with poisoning because fewer toxicology beds were available. In the first 10 weeks of the pandemic, we found no evidence that occupancy rates differed compared with the same days in 2019 (appendix 3 p 2). It is against hospital policy to turn away patients who need admission—if capacity is reached, patients are admitted as ‘floor' patients and allocated to wards. We found evidence this occurred during early June, 2020, suggesting that ward capacity is unlikely to have been a significant contributor to our findings. Fourth, we could not accommodate seasonal trends in our models as we were only able to collect data for 15 months before the pandemic. However, comparison with March, 2019, did not suggest that any of the changes observed following the onset of lockdown in March, 2020, could be due to seasonal effects. Fifth, our analysis is restricted to a single hospital in the Kandy district, and known regional variations in suicidal behaviour in Sri Lanka^38^, ^39^ mean that our findings might not apply to other districts. Last, the results referred to here are restricted to the impact of COVID-19 during the early months of the pandemic. As the effects of the initial lockdown measures are realised and the pandemic continues, it will be important to continue surveillance to assess the longer term consequences of the pandemic and the lockdown, such as socioeconomic repercussions.

The findings of this study present a reassuring picture of a lower incidence of self-poisoning presentations to one hospital in a LMIC immediately following the start of the lockdown, over and above a longer-term decline in hospital presentations for self-poisoning. The reduction appears to be due to a reduction in medicinal overdoses. Some caution is required in case individuals who have self-harmed were less likely to present to hospital after self-poisoning because of fears over COVID-19. Future research should investigate this and public health messaging should emphasise the importance of seeking help early.

**This online publication has been corrected. The corrected version first appeared at thelancet.com/psychiatry on September 17, 2021**

## Data sharing

Data are available in the University of Bristol (UK) data repository. Given the sensitivity of the data, only researchers at verifiable institutions will be able to access data. Any requests will be reviewed by the University of Bristol Access Committee, which includes senior researchers and representatives from the university. Data will only be released once a controlled data access agreement has been signed by a nominated institutional signatory. A data dictionary will also be made available.

## Declaration of interests

We declare no competing interests.

## Supplementary Materials

Sinhalese translation of the abstract

Tamil translation of the abstract
