# Correction to *Lancet Psychiatry* 2021; 8: 892–900

**DOI:** 10.1016/S2215-0366(21)00388-6

**Published:** 2021-11

*Knipe D, Silva T, Aroos A, et al. Hospital presentations for self-poisoning during COVID-19 in Sri Lanka: an interrupted time-series analysis.* Lancet Psychiatry *2021; **8:** 892–900*. https://doi.org/10.1016/S2215-0366(21)00242-X—In this Article, in figure 1, the observed and expected lines have been corrected. This correction has been made to the online version as of Sept 17, 2021.

