# Supplementary material for: Hospital presentations for self-poisoning during COVID-19 in Sri Lanka: an interrupted time-series analysis
Source: Lancet Psychiatry. 2021 Oct;8(10):892–900. doi: 10.1016/S2215-0366(21)00242-X (PMC8445699; doi:10.1016/S2215-0366(21)00242-X)
Supplement: Sinhalese translation of the abstract [file mmc1.pdf]

Supplementary appendix 1

This translation in Sinhala was submitted by the authors and we reproduce it as supplied. It has not been peer reviewed. *The Lancet's* editorial processes have only been applied to the original in English, which should serve as reference for this manuscript.

මෙම සිංහල භාෂා පරිවර්තනය කතුවරුන් විසින් යොමුකරන ලද අතර අපි එය සපයා ඇති පරිදි ඉදිරිපත් කරන්නෙමු. මේ සඳහා තුල්‍යසමාලෝචනයක් සිදුකර නොමැත. ලැන්සට්හි සංස්කරණ ක්‍රියාවලීන් යෙදී ඇත්තේ මේ සඳහා යොමු කිරීමක් වන මෙහි ඉංග්‍රීසි මුල් පිටපතට පමණි.

Supplement to: Knipe D, Silva T, Aroos A, et al. Hospital presentations for self-poisoning during COVID-19 in Sri Lanka: an interrupted time-series analysis. *Lancet Psychiatry* 2021; published online July 29. http://dx.doi.org/10.1016/S2215-0366(21)00242-X.

සාරාංශ

පර්යේෂණයේ පසුබිම: COVID-19 හා සම්බන්ධ මහජන සෞඛ්‍ය ක්‍රියාමාර්ග, මානසික සෞඛ්‍යයට සහ සියදිවි හානි කරගැනීම පිළිබඳව බලපාන ආකාරය ගැන පුළුල් අවධානයක් වර්තමානයේ යොමු වී ඇත. නමුත් සියදිවි හානි කරගැනීම් සහ සියදිවි හානි කරගැනීමට උත්සහ කිරීම් වල බලපෑම වැඩිම වන, අඩු මධ්‍යම ආදායම් ලබන රටවලින් මේ පිළිබඳව ලැබී ඇති යුතු දත්ත හිඟ වේ.

පර්යේෂණය සිදුකල ක්‍රමය: රෝහලට ඉදිරිපත් වන, ස්වයං විෂ වීම් මගින් සියදිවි හානි කරගැනීමට උත්සහ කල සිද්ධීන් වලට, වසංගත තත්වය හේතුවෙන් ඇති වී ඇති බලපෑම පිළිබඳව ගවේෂණය කිරීමේ අරමුණ ඇතිව, අප විසින් ශ්‍රී ලංකාවේ පේරාදෙණිය ශික්ෂණ රෝහලේ ස්වයං විෂ වීම් කරගැනීමට උත්සහ කල සිද්ධීන් පිලිබඳ තොරතුරු රැස් කිරීමේ නව ලේඛනයක් ස්ථාපිත කරන ලදී. අදාළ වාටුවට ඇතුලත් වූ රෝගීන් අතර ස්වයං විෂ වීම් කරගැනීමට උත්සහ කල සිද්ධීන් හඳුනා ගන්නා ලදී. 2019 ජනවාරි 01 වන දින සිට 2020 අගෝස්තු 31 වන දින දක්වා, ස්වයං විෂ වීම් කරගැනීමට උත්සහ කරගැනීම නිසා, පේරාදෙණිය ශික්ෂණ රෝහලේ ඇතුලත්වී ප්‍රතිකාර ලැබූ රෝගීන්ට අදාල නිර්නාමික දත්ත එකතු කරන ලදී. වාට්ටුවට ඇතුළත් වූ දිනය, වයස, ස්ත්‍රී පුරුෂ භාවය සහ ස්වයං විෂවීම සඳහා භාවිතා කල ක්‍රමය පිළිබඳ දත්ත එක්රැස් කරන ලදී. සතිපතා ස්වයං විෂ වීම් කරගැනීමට උත්සහ කර රෝහලට පැමිණෙන සිද්ධීන් වලට COVID-19 වසංගතයේ නිසා ඇති වූ බලපෑම සමස්ථයක් වශයෙන් සහ වයස සහ ස්ත්‍රී පුරුෂ භාවය අනුව ආදර්ශනය කිරීම සඳහා අප බාධාකාරී කාල ශ්‍රේණි විශ්ලේෂණ ක්‍රමය (interrupted

time-series analysis) භාවිතා කරන ලදී. වාට්ටුවට ඇතුලත්වූ දිනය සඳහන් වී නොමැති පුද්ගලයන් ප්‍රධාන විශ්ලේෂණයෙන් බැහැර කර ඇත.

පර්යේෂණ ප්‍රතිඵල: පිරිමි 584/1410 (42%) සහ කාන්තාවන් 761/1410 (54%) ස්වයං විෂවීම් කිරීම හේතුවෙන් රෝහලට ඉදිරිපත් වී ඇත. වසංගතයට පෙර ප්‍රවණතාවන්ට සාපේක්ෂව, වසංගත කාල පරිච්ඡේදයේදී ස්වයං විෂවීම් කරගැනීමට උත්සහ කිරීම හේතුවෙන් රෝහලට පැමිණීමේ 32% ක අඩුවීමක් (95% CI 12%, 48%) නිරීක්ෂණය විය. වසංගතයේ බලපෑම ස්ත්‍රී පුරුෂභාවය (rate ratio 0·63, 95% CI 0·44–0·94, for females vs 0·85, 0·57–1·26, for males; $P_{interaction}$=0·43) හෝ වයස (0·63, 95% CI 0·44–0·93, for patients aged <25 years vs 0·81, 0·57–1·16, for patients aged ≥25 years; $P_{interaction}$=0·077) අනුව වෙනස් වූ බවට සාක්ෂියක් අපට හමු නොවීය.

නිගමනය: මාරාන්තික නොවන සියදිවි හානි කරගැනීම් වලට COVID-19 වසංගතයේ බලපෑම තක්සේරු කිරීම සඳහා, අඩු මධ්‍යම ආදායම් ලබන රටක් මගින් සිදු කරන ලද පළමු අධ්‍යයනය මෙය වේ. වසංගතය අතරතුර රෝහලට ඉදිරිපත් වන ස්වයං විෂවීම් කරගැනීමට උත්සහ කල සිද්ධි පහත වැටීමෙන් පිළිබිඹු වන්නේ, ස්වයංවිෂවීම් කරගැනීමට උත්සහ කල සිදුවීම්වල සැබෑ අඩුවීමක් නොව, ස්වයං විෂ වීම් කර ගැනීමට උත්සහ කල පුද්ගලයන් වෛද්‍ය ප්‍රතිකාර ලබා ගැනීමට රෝහලට ඉදිරිපත් වීම් පිළිබඳව අඩුවක් නම්, මහජන සෞඛ්‍ය පණිවුඩ මගින් පළමුඅවස්ථාවේදීම උපකාර පැතීමේ වැදගත්කම අවධාරණය කළ යුතුය.
