# Supplementary material for: Hospital presentations for self-poisoning during COVID-19 in Sri Lanka: an interrupted time-series analysis
Source: Lancet Psychiatry. 2021 Oct;8(10):892–900. doi: 10.1016/S2215-0366(21)00242-X (PMC8445699; doi:10.1016/S2215-0366(21)00242-X)
Supplement: Tamil translation of the abstract [file mmc2.pdf]

THE LANCET
Psychiatry

Supplementary appendix 2

This translation in Tamil was submitted by the authors and we reproduce it as supplied. It has not been peer reviewed. *The Lancet's* editorial processes have only been applied to the original in English, which should serve as reference for this manuscript.

தமிழில் இந்த மொழிபெயர்ப்பு ஆசிரியர்களால் சமர்ப்பிக்கப்பட்டது, நாங்கள் அதை வழங்கியபடி மீண்டும் உருவாக்குகிறோம்.இது மதிப்பாய்வு செய்யப்படவில்லை. லான்செட்டின் தலையங்க செயல்முறைகள் ஆங்கிலத்தில் அசலுக்கு மட்டுமே பயன்படுத்தப்பட்டுள்ளன, இது இந்த கையெழுத்துப் பிரதிக்கான குறிப்பாக செயல்பட வேண்டும்.

Supplement to: Knipe D, Silva T, Aroos A, et al. Hospital presentations for self-poisoning during COVID-19 in Sri Lanka: an interrupted time-series analysis. *Lancet Psychiatry* 2021; published online July 29. http://dx.doi.org/10.1016/S2215-0366(21)00242-X.

சுருக்கம்

பின்னணி:

Covid19 தொற்றுடன் சம்பந்தப்பட்ட பொது சுகாதார நடவடிக்கைகளின் மூலம் ஏற்பட்டுள்ள மனநல தாக்கம் உட்பட சுய தீங்கு பற்றி பரவலான அக்கறை காணப்படுகிறது. தற்கொலை மற்றும் சுய தீங்கு அதிகமாக காணப்படும் குறைந்த மற்றும் நடுத்தர வருமானம் உடைய நாடுகளிடையே அது சம்பந்தப்பட்ட உயர்தரமான ஆதாரங்கள் குறைவாகவே காணப்படுகிறது. இவ்வாய்வின் நோக்கு சுய நச்சு மூலமான மருத்துவமனை அனுமதிகளினிடையே பெருந்தொற்றின் தாக்கத்தை அறிவதாகும்.

ஆய்வு முறை:

இந்த குறுக்கிடப்பட்ட நேர-தொடர் பகுப்பாய்வில் நாங்கள் குறைந்த மற்றும் நடுத்தர வருமானம் உடைய நாடான இலங்கையின் பேராதனை வைத்தியசாலையில் சுய தீங்கு (சுய நச்சு எடுத்தல்) காரணமாக அனுமதிக்கப்பட்ட நோயாளர்களினிடையே ஒரு புதிய பதிவு புத்தகம் ஒன்றை நிறுவினோம். ஒரு நிலையான பதிவுத்தாளைக் கொண்டு நச்சுயியல் வாட்டில் 2019 ஜனவரி 1 முதல் 2020 ஆகஸ்ட் 31 வரை சுய நச்சு சம்பந்தமாக அனுமதிக்கப்பட்டவர்கள் பற்றிய தரவுகளை சேகரித்தோம். சிகிச்சையளிக்கும் மருத்துவரால் சுய நச்சு எடுத்தவர்கள் என அடையாளம் காணப்பட்டவர்கள் மட்டுமே இவ்வாய்வில் சேர்த்துக்கொள்ளப்பட்டனர். அனுமதிக்கப்பட்ட திகதி, வயது அல்லது பிறந்த திகதி , பால் மற்றும் சுய நச்சு முறை பற்றிய தகவல்களை சேகரித்தோம். இனம் குறித்த தரவு எதுவும் கிடைக்கவில்லை.COVID-19 பெருந்தொற்றின் தாக்கத்தை தரவுபடுத்தும் முகமாக குறுக்கிடப்பட்ட நேர-தொடர் பகுப்பாய்வை பயன்படுத்தி தொற்றுக்கு முன் (Jan 1, 2019–March 19, 2020) மற்றும் தொற்றின் போதான (March 20–Aug 31, 2020) சுயநச்சு சம்பந்தப்பட்ட வாராந்த மருத்துவமனை அனுமதிகள், ஒட்டுமொத்த தரவுகள்,வயது(<25 வயது vs ≥25 வயது) மற்றும் பால் போன்ற தரவுகளால் ஆராய்ந்தோம். அனுமதிக்கப்பட்ட திகதி பற்றிய தரவுகள் அற்றவர்கள் இவ்வாய்விலிருந்து விலக்கப்பட்டனர்.

முடிவுகள்: ஜனவரி 1, 2019 மற்றும் ஆகஸ்ட் 31, 2020 க்கு இடையில், 1401 நபர்கள் (584 [41·7%] ஆண்கள், 761 [54·3%] பெண்கள், மற்றும் 56 [4·0%] பாலினம் அறியப்படாதவர்கள்) சுயநச்சு சம்பந்தமாக வைத்திய சாலையில் அனுமதிக்கப்பட்டிருந்ததோடு அவர்கள் அனுமதிக்கப்பட்ட திகதி பற்றிய தரவுகளும் இருந்தன. Covid 19 பெருந்தொற்றுக்கு முற்பட்ட காலப்போக்குடன் ஒப்பிடும்போது பெருந்தொற்றுடைய காலத்தில் சுய நச்சு சம்பந்தப்பட்ட மருத்துவமனை அனுமதிகளில் 32% குறைவு (95% CI 12-48) காணப்பட்டது (வீத விகிதம்(rate ratio) 0·68, 95% CI 0·52–0·88; p=0·0032). பெருந்தொற்றின் தாக்கம் பால்(வீத விகிதம் (rate ratio) 0·63, 95% CI 0·44–0·94, பெண்கள் vs 0·85, 0·57–1·26, ஆண்கள்; $P_{interaction}$=0·43) மற்றும் வயது (0·63, 95% CI 0·44–0·93, <25 வயது vs 0·81, 0·57–1·16, ≥25 வயது; $P_{interaction}$=0·077) போன்றவற்றோடு வேறுபடுகிறது என்பதற்கான எந்த ஆதாரமும் எங்களுக்கு கிடைக்கவில்லை.

முடிவுரை:சுயதீங்கின் (உயிராபத்து அற்ற) அடிப்படை போக்கில் தொற்றுநோயின் தாக்கத்தை மதிப்பிடுவதற்கான குறைந்த- நடுத்தர வருமான நாடுகளின் முதல் ஆய்வு இதுவாகும்.பெரும் தொற்று நிலையின் போது மருத்துவமனையில் அனுமதிக்கப்பட்டவர்களின் வீழ்ச்சி உண்மையான வீழ்ச்சி அல்லாது சுய நச்சு எடுத்த நபர்கள் மருத்துவ சிகிச்சையை நாடுவதில் உள்ள குறைவை பிரதிபலிக்கின்றது என்றால், உடனடி மருத்துவ உதவியை நாடுவதின் முக்கியத்துவத்தை பொது சுகாதார அறிவுறுத்தல்கள்களின் மூலம் வலியுறுத்துவது அவசியமாகும்.
