# Supplementary material for: Hospital presentations for self-poisoning during COVID-19 in Sri Lanka: an interrupted time-series analysis
Source: Lancet Psychiatry. 2021 Oct;8(10):892–900. doi: 10.1016/S2215-0366(21)00242-X (PMC8445699; doi:10.1016/S2215-0366(21)00242-X)
Supplement: Supplementary appendix 3 [file mmc3.pdf]

THE LANCET
Psychiatry

Supplementary appendix 3

This appendix formed part of the original submission and has been peer reviewed. We post it as supplied by the authors.

Supplement to: Knipe D, Silva T, Aroos A, et al. Hospital presentations for self-poisoning during COVID-19 in Sri Lanka: an interrupted time-series analysis. *Lancet Psychiatry* 2021; published online July 29. http://dx.doi.org/10.1016/S2215-0366(21)00242-X.

Supplementary methods

Example of search terms used for our living systematic review.

Search terms for PubMed

((selfharm*[TIAB] OR self-harm*[TIAB] OR selfinjur*[TIAB] OR self-injur*[TIAB] OR selfmutilat*[TIAB] OR self-mutilat*[TIAB] OR suicid*[TIAB] OR parasuicid*[TIAB) OR (suicide[TIAB] OR suicidal ideation[TIAB] OR attempted suicide[TIAB]) OR (drug overdose[TIAB] OR self?poisoning[TIAB]) OR (self-injurious behavio?r[TIAB] OR self?mutilation[TIAB] OR automutilation[TIAB] OR suicidal behavio?r[TIAB] OR self?destructive behavio?r[TIAB] OR self?immolation[TIAB])) OR (cutt*[TIAB] OR head?bang[TIAB] OR overdose[TIAB] OR self?immolat*[TIAB] OR self?inflict*[TIAB]))) AND ((coronavirus disease?19[TIAB] OR sars?cov?2[TIAB] OR mers?cov[TIAB]) OR (19?ncov[TIAB] OR 2019?ncov[TIAB] OR n?cov[TIAB]) OR ("severe acute respiratory syndrome coronavirus 2" [Supplementary Concept] OR "COVID-19" [Supplementary Concept] OR COVID-19 [tw] OR coronavirus [tw] OR nCoV[TIAB] OR HCoV[TIAB] OR ((virus*[Title] OR coronavirus[Title] OR nCoV[Title] OR infectious[Title] OR HCoV[Title] OR novel[Title])AND (Wuhan[Title] OR China[Title] OR Chinese[Title] OR 2019[Title] OR 19[Title] OR COVID*[Title] OR SARS-Cov-2[Title] OR NCP*[Title]) OR “Coronavirus”[MeSH]))))

Additional post-hoc analysis:

There were two changes during the pandemic which may have impacted on data collection or reporting which may have contributed to any changes observed. The first change relates to the change in the physical location of the ward on the 26th March 2020, which resulted in the admission books related to admissions pre-May 2020 remaining in the original location. This meant that the study team were unable to trace basic data for all admissions pre-May 2020 when the hospital bed-head ticket was missing. We, therefore, tested the robustness of our findings by adding an additional interruption to the model in the form of a binary coded variable indicating the time period before and after May 2020. The second change relates to the change in the way data were recorded by the study team. Pre-June 2020 data were collected from patient records after the patient was discharged from hospital care (i.e. retrospective data collection). After June 2020 we collected data prospectively from patients who were admitted to the ward. As with the previous change, we re-fitted our main analysis model with an additional interruption. The binary coded variable indicated whether the time period was during prospective (Jun-Aug 2020) or retrospective (Jan 2019 – May 2020) data collection.

Supplementary results

Supplementary figure 1 – Changes in hospital presenting self-poisoning by medicine type following the COVID-19 pandemic in Teaching Hospital Peradeniya, Sri Lanka

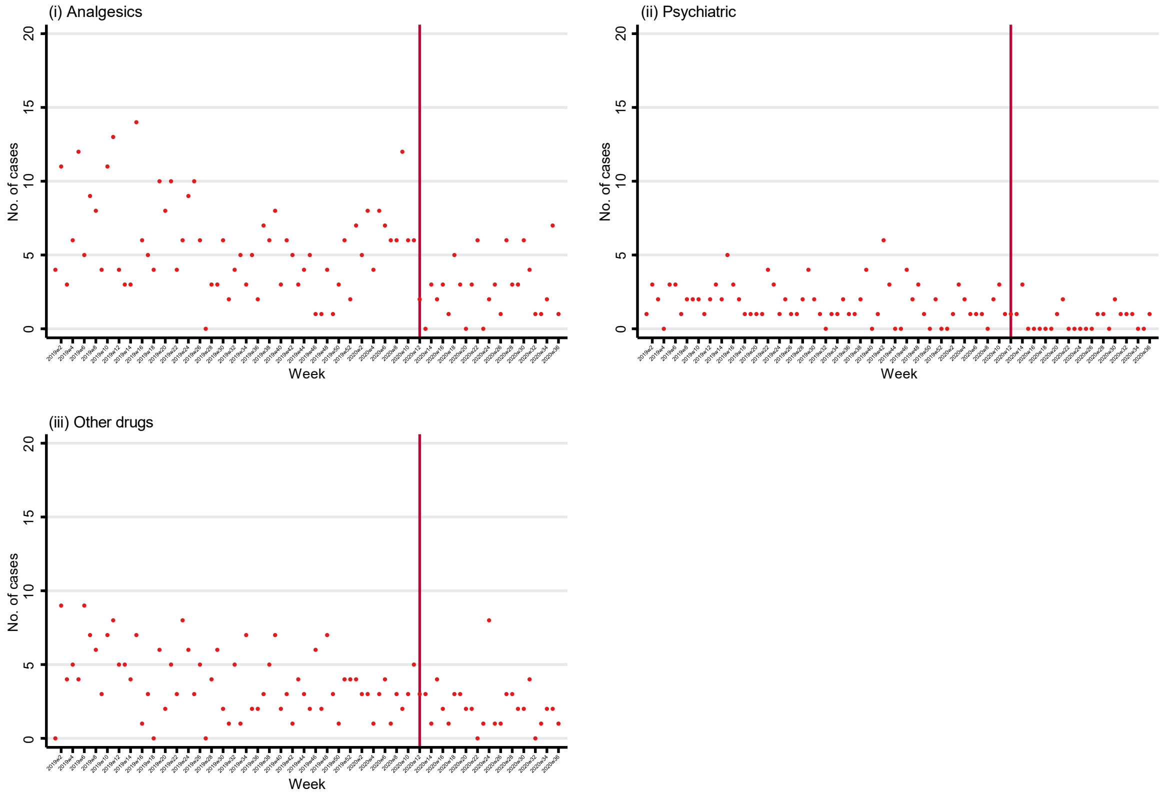

Red line indicates 20/03/2020 national lockdown

Additional post-hoc analysis:

The addition of an extra interruption in May 2020 to our main time series model found a consistent drop in the number of presentations during the pandemic period (RR 0.58 95% CI 0.37, 0.89), with no statistical evidence of a May 2020 interruption (p=0.36). A similar drop in the pandemic period (RR 0.65 95% CI 0.47, 0.91) was observed when we re-fitted our main time series model with a June 2020 interruption to indicate the change in data collection from retrospective to prospective data collection. We found no statistical evidence of an interruption in June 2020 (p=0.74).

We also compared the percentage of hospital admissions to the toxicology ward on 10 randomly selected days from each of the 10 weeks following the pandemic to the same days in 2019. There was no evidence that the ward reached its full capacity on any of the days in either 2019 or 2020. On average ward occupancy was similar in 2019 (44.7% (95% CI 38.6%, 50.8%)) and 2020 (37.2%, 95% CI 30.0%, 44.5%).

Supplementary table 1 – Comparison of bed occupancy between 2019 and 2020

			2019 (total beds 36)			2020 (total beds 18)		
Month	Week	Day	Date	Ward Total	% occupied	Date	Ward Total	% occupied
March	5	Monday	18/03/2019	16	44.4	23/03/2020	9	50.0
March	6	Tuesday	26/03/2019	18	50.0	30/03/2020	6	33.3
April	1	Wednesday	03/04/2019	13	36.1	04/01/2020	8	44.4
April	2	Thursday	11/04/2019	15	41.7	04/09/2020	8	44.4
April	3	Friday	19/04/2019	15	41.7	17/04/2020	7	38.9
April	4	Saturday	27/03/2019	9	25.0	25/04/2020	5	27.8
May	1	Sunday	05/05/2019	18	50.0	05/03/2020	6	33.3
May	2	Monday	06/05/2019	22	61.1	05/04/2020	7	38.9
May	3	Tuesday	14/05/2019	16	44.4	05/12/2020	2	11.1
May	4	Wednesday	22/05/2019	19	52.8	20/05/2020	9	50.0
